# Ischemic Stroke as an Initial Manifestation of Antiphospholipid Syndrome in an Adolescent: A Case Report

**DOI:** 10.7759/cureus.55579

**Published:** 2024-03-05

**Authors:** Jimena Gonzalez-Salido, Natalia M Barron-Cervantes, Jimena Colado-Martinez, Sara Fernanda Arechavala Lopez, Virna L Mosqueda-Larrauri, Juan L Ortiz-Herrera, Enrique Piña-Rosales, Javier Martinez-Bautista

**Affiliations:** 1 Medicine, Universidad La Salle, Mexico City, MEX; 2 School of Medicine, Universidad Panamericana, Mexico City, MEX; 3 School of Medicine, Universidad La Salle, Mexico City, MEX; 4 Medicine, Universidad Autónoma Metropolitana, Mexico City, MEX; 5 School of Medicine, Universidad Nacional Autónoma de México, Mexico City, MEX; 6 Internal Medicine, Fundación Clínica Médica Sur, Mexico City, MEX; 7 Neurology, Fundación Clínica Médica Sur, Mexico City, MEX

**Keywords:** childhood stroke, adolescent stroke, ischemic stroke, antiphospholipid syndrome, cerebrovascular accident, pediatric stroke

## Abstract

Cerebrovascular diseases in pediatric patients are relatively rare. Ischemic stroke in adolescents is associated with a poor prognosis. The most common causes include systemic diseases, such as heart disease and hypercoagulation disorders. It is important to mention that one of the most common acquired hypercoagulation states is the antiphospholipid syndrome (APS). Patients with this disease may present stroke as the first clinical manifestation, which not only increases morbidity in these patients but presents a diagnostic challenge. This case presents one example of how APS can present as a pediatric stroke. The diagnostic approach should always be through the presence of specific antibodies accompanied by the presence of a thromboembolic episode proven by catheterization or an imaging study. In the brain, the preferred imaging study is magnetic resonance imaging. Management is based on anticoagulation therapy and continuous monitoring in the intensive care unit.

## Introduction

Ischemic stroke (IS) is one of the most uncommon clinical pictures presented in the pediatric population, thus it remains a diagnostic challenge. It affects an estimated one to two per 100,000 children (non-neonates) annually in Western developed countries with a similar incidence of hemorrhagic stroke (HS) of 1-1.7 in 100,000 [1]. Cerebrovascular disease is a serious neurological disorder in children associated with poor prognosis and high morbidity and mortality rates. Even as an acute onset, delays in diagnosis associated with low diagnostic suspicion are common, which imitates the opportunity for an early intervention. A thorough investigation is required for all cases, which includes neuroimaging and laboratory testing. MRI is the study of choice in all types of pediatric IS as early changes can be detected through diffusion-weighted imaging [2]. Laboratory investigations should always be focused on ruling out systemic causes, such as cardiovascular disease or hypercoagulable states, since the patient's prognosis will always be associated with this. One of the most common causes is autoimmune diseases, such as antiphospholipid syndrome (APS) [3]. One of the main problems with these diseases is that they are not usually considered as a differential diagnosis in the pediatric population and even less in the male population since they are pretty uncommon. The following review presents the case of a 15-year-old male patient who presented with sudden onset of left-sided fascio-corporal hemiparesis, dysarthria, limiting mobility, and loss of urinary sphincter control in a first-level private surgical center in Mexico City and shows how it was proven that these symptoms were caused because of a pediatric IS that was presented as the first clinical manifestation of APS. This case is presented to further expand the knowledge about pediatric IS, as well as highlighting the importance of looking for systemic diseases that may be causing this clinical manifestation, and also to highlight the uniqueness of this case presentation in the pediatric population with only a few cases described in the literature.

## Case presentation

A previously healthy 15-year-old Latin American male patient presented to the emergency room (ER) with sudden onset of left-sided fascio-corporal hemiparesis, left eye ptosis, dysarthria, limiting mobility, and loss of urinary sphincter control once. He had partial recovery at the time of admission. During his stay in the ER, the patient mentioned that 24 hours earlier, he presented a similar episode where mobility was nearly fully restored minutes after the event. However, this episode was not self-limited, leading him to seek medical evaluation. Also, no loss of consciousness was presented. As relevant hereditary and familial history, it was mentioned that the mother experienced HELLP (hemolysis, elevated liver enzymes, and low platelets) syndrome at the end of her pregnancy, a half-brother on the maternal side passed away due to schizophrenia, and the maternal grandmother had a history of thrombosis in her left leg. No family history of consanguineous marriage was presented in this case. Upon admission, the neurological physical examination revealed the patient to be conscious, awake, and responsive. There was evidence of left-sided pyramidal syndrome. Cranial nerve examination indicated isochoric and normoreflective pupils and no ptosis presented, with other cranial nerves exhibiting normal function. The strength in the upper extremities was 5/5, while in the lower extremities was 4/5, with preserved tone and tropism. Reflexes were 3/4 in the lower and 2/4 in the upper extremities. Babinski reflex was present on the left side. Exteroceptive sensitivity was found to be within normal limits, and there were no sensory disturbances. Cerebellar signs were absent and coordination was normal, gait was only affected by hemiparesis. His laboratory studies showed within normal parameters (Table 1).

**Table 1:** Initial laboratory tests Initial laboratory tests included a complete blood count (CBC), blood chemistry test (BCT), complement components, inflammatory markers, specific coagulation proteins, and specific antibodies for antiphospholipid syndrome and vasculitis. APTT: activated partial thromboplastin time; PT: prothrombin time; dRVVT: dilute Russell viper venom test; C-ANCA: cytoplasmic antineutrophil cytoplasmic antibodies; P-ANCA: perinuclear antineutrophil cytoplasmic antibodies.

Parameter		Value	Reference values
Hemoglobin		15.7 g/dL	12-18 g/dL
Hematocrit		47.3%	36-48%
Platelets		194 x 10^3^/uL	150-450 x 10^3^/uL
Leukocyte count		7.3 x 10^3^/uL	4.5-11 x 10^3^/uL
Absolute neutrophils		4 x 10^3^/uL	2.5-7 x 10^3^/uL
Absolute lymphocytes		2.6 x 10^3^/uL	1-4 x 10^3^/uL
Absolute monocytes		0.6 x 10^3^/uL	2-4 x 10^3^/uL
Absolute eosinophils		0.2 x 10^3^/uL	3-3.5 x 10^3^/uL
Fibrinogen		244 mg/dL	177-410 mg/dL
Ferritin		50 ng/mL	24-336 ng/mL
Prothrombin		11.4 seg	9.8-12.5 seg
Thromboplastin		28.5 seg	24.5-32.0 seg
Glucose		99 mg/dL	72-99 mg/dL
Creatinine		0.76 mg/dL	0.31-1.00 mg/dL
Blood urea nitrogen (BUN)		12.8 mg/dL	7-22 mg/dL
Uric acid		5.8 mg/dL	3.5-7.2 mg/dL
D-dimer		270 μg/l	0.00-499.00 μg/l
Erythrocyte sedimentation rate		3 mm/h	0-10 mm/h
Procalcitonin		<0.02 ng/mL	0.00-0.06 ng/mL
Factor VIII		193 IU/dl	70-150 IU/dl
Protein S (total)		79 U/dL	70-140 U/dL
Protein S (free)		102 U/dL	57-171 U/dL
Protein C antigen		102 U/dL	72-160 U/dL
C3		121.20 mg/dL	79-152 mg/dL
C4		24.80 mg/dL	16.0-38.0 mg/dL
Beta 2 glycoprotein IgG		<6.4 CU	<20 CU
Beta 2 glycoprotein IgM		<1.1 CU	<20 CU
Beta 2 glycoprotein IgA		<4.0 CU	<20 CU
Cardiolipin antibody IgG		8.1 CU	<20 CU
Cardiolipin antibody IgM		1.5 CU	<20 CU
Lupus anticoagulant APTT		35.6 seconds	24.5-32 seconds
Lupus anticoagulant PT		16.7 seconds	10-13.7 seconds
Lupus anticoagulant dRVVT		47.7 seconds	31-44 seconds
C-ANCA		Negative	
P-ANCA		Negative	

**Figure 1:**
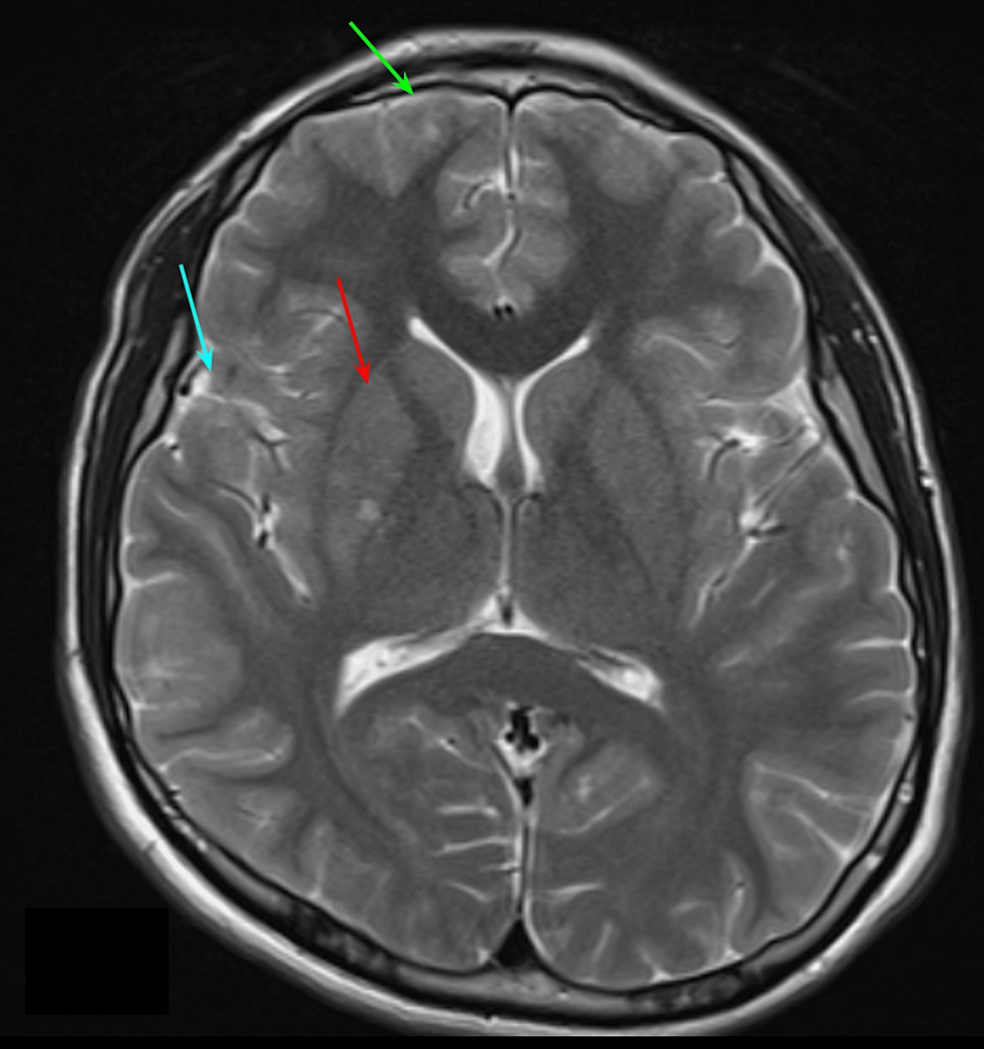
First brain MRI T2 image Hyperacute ischemic vascular disease in the right middle frontal (green arrow) and temporal gyri (blue arrow) with ipsilateral lentiform nucleus involvement (red arrow).

**Figure 2:**
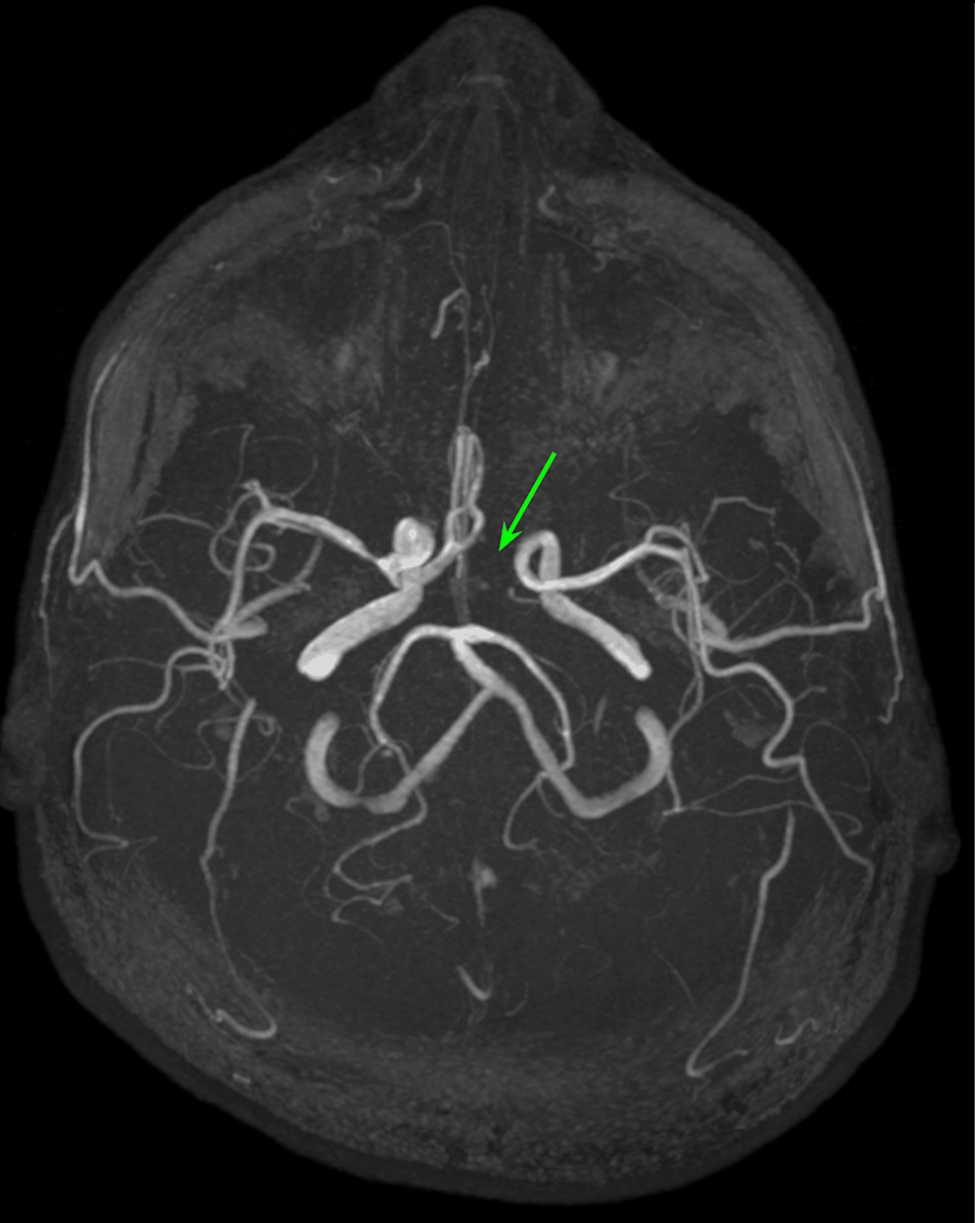
Cerebral magnetic resonance angiography Focal intracranial vasculopathy related to a distal thrombus and agenesis of the A1 segment of the left anterior cerebral artery (green arrow).

Brain MRI revealed hyperintense areas on diffusion-weighted imaging (DWI) and correlative hypointense areas on apparent diffusion coefficient (ADC) maps in the right middle frontal and temporal gyri with ipsilateral lentiform nucleus involvement with a focal area of stenosis at the emergence of the right middle cerebral artery (M1 segment), and apparent agenesis of the A1 segment of the left anterior cerebral artery was also noted (Figure 1). Cerebral magnetic resonance angiography (MRA) confirmed focal intracranial vasculopathy related to a distal thrombus (Figure 2).

**Figure 3:**
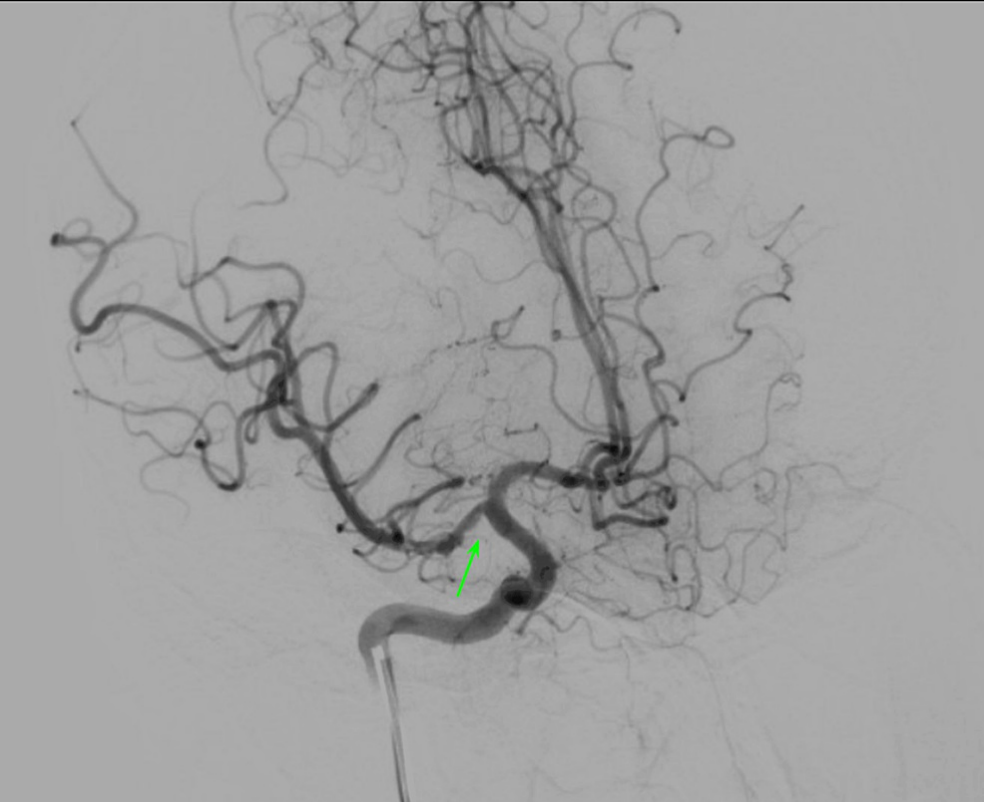
Cerebral angiography Thrombus located at the emergence of the right middle cerebral artery, M1 segment (green arrow).

The echocardiogram indicated no patent foramen ovale (PFO) and a normal left ventricular ejection fraction (73%) with normal valves. Electroencephalogram (EEG) findings were abnormal, showing increased cortical excitability in the left frontotemporal region. Immunological profiling was requested and it revealed the presence of a positive lupus anticoagulant. The patient was initiated with anticoagulation with enoxaparin 60 mg subcutaneous (SC) every 12 hours and acetylsalicylic acid 100 mg per oral (PO) every 24 hours. The patient underwent diagnostic angiography, during which the presence of a thrombus was confirmed at the emergence of the right middle cerebral artery (Figure 3). No therapeutic intervention was administered due to the temporal constraints observed between the onset of symptoms and the angiographic procedure.

Due to sinus bradycardia and the need for post-invasive procedure care, the patient was kept under observation in the ICU and hemodynamic monitoring for 24 hours. He showed favorable progress with no new focal deterioration events and normalization of vital signs. The transition from systemic anticoagulation with enoxaparin to acenocoumarol was carried out, with target international normalized ratio (INR) levels being monitored. Hydroxychloroquine and aspirin were also initiated. Due to satisfactory clinical improvement, it was decided to discharge the patient for further follow-up in an outpatient setting. After 12 weeks post-discharge, subsequent laboratory examinations conducted externally revealed no anomalies in the laboratory tests (Table 2). Additionally, the patient continued to experience positive physical recovery, slowly gaining strength and mobility on the left side.

**Table 2:** Twelve weeks post-discharge laboratory tests Control laboratory tests included complete blood count (CBC), blood chemistry test (BCT), complement components, and specific antibodies for antiphospholipid syndrome. APTT: activated partial thromboplastin time; PT: prothrombin time; dRVVT: dilute Russell viper venom test.

Parameter		Value	Reference values
Hemoglobin		16.4 g/dL	12-18 g/dL
Hematocrit		47.7%	36-48%
Platelets		244 x 10^3^/uL	150-450 x 10^3^/uL
Leukocyte count		5.34 x 10^3^/uL	4.5-11 x 10^3^/uL
Absolute neutrophils		2.31 x 10^3^/uL	2.5-7 x 10^3^/uL
Absolute lymphocytes		2.33 x 10^3^/uL	1-4 x 10^3^/uL
Absolute monocytes		0.29 x 10^3^/uL	2-4 x 10^3^/uL
Absolute eosinophils		0.32 x 10^3^/uL	3-3.5 x 10^3^/uL
Prothrombin		39.7 seg	9.8-12.5 seg
Thromboplastin		68.4 seg	24.5-32.0 seg
Glucose		90 mg/dL	72-99 mg/dL
Creatinine		0.79 mg/dL	0.31-1.00 mg/dL
Blood urea nitrogen (BUN)		12.5 mg/dL	7-22 mg/dL
Uric acid		5.8 mg/dL	3.5-7.2 mg/dL
C3		103 mg/dL	79-152 mg/dL
C4		21.8 mg/dL	10-40 mg/dL
Beta 2 glycoprotein IgG		<6.4 CU	<20 CU
Beta 2 glycoprotein IgM		<1.1 CU	<20 CU
Beta 2 glycoprotein IgA		<4.0 CU	<20 CU
Cardiolipin antibody IgG		6.1 CU	<20 CU
Cardiolipin antibody IgM		<1 CU	<20 CU
Lupus anticoagulant APTT		25.6 seconds	24.5-32 seconds
Lupus anticoagulant PT		12.3 seconds	10-13.7 seconds
Lupus anticoagulant dRVVT		41.2 seconds	31-44 seconds

**Figure 4:**
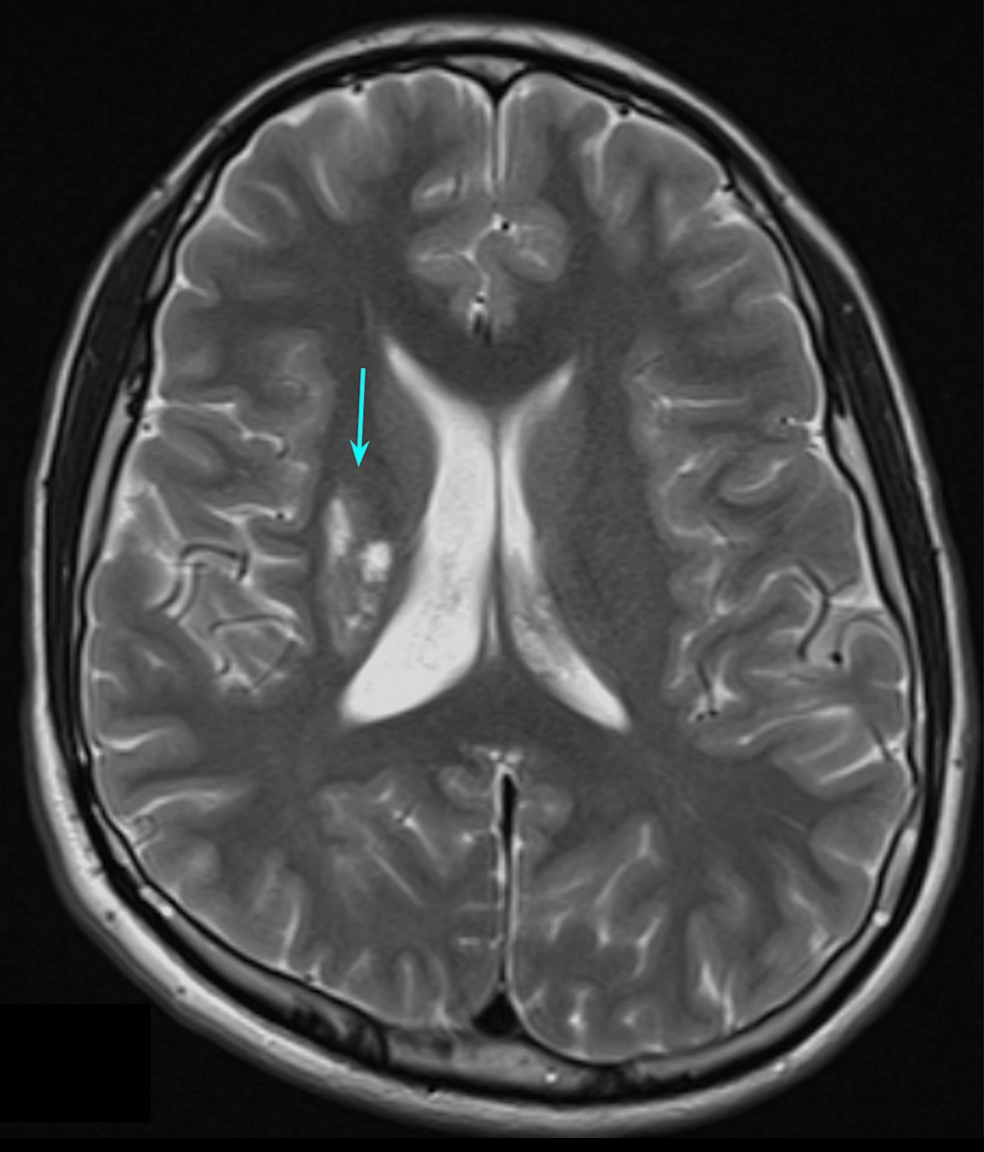
Second brain MRI T2 image Chronic changes associated with the ischemic process, with major involvement of the lentiform nucleus (blue arrow).

A new MRI (Figure 4) was performed two months after the event where chronic changes associated with the ischemic process can be observed, with major involvement of the lentiform nucleus.

## Discussion

Cerebrovascular diseases can be presented as HS and IS. Even though IS is the most common in both the pediatric and adult population, HS in children is closer to 50%. IS is rarely presented in the pediatric population; however, its presence is always associated with a poor prognosis. Incidence in the US is estimated to be 0.5 to 2.4 cases per 100,000 births per year. Of all cases, the most common cause is perinatal stroke, with an incidence of one per 2,500 births per year. Systemic disease is the most common cause of stroke in kids. Hypercoagulation states, hematologic diseases, and cardiac malformations are the most common causes presented. Individualizing the case is really important, as some diseases present more in different kinds of populations. For example, stroke secondary to sickle cell disease is more common in African American children. Pathophysiology results from an occlusion in a cerebral artery, the most common being the middle cerebral artery (MCA). According to its location, clinical patterns may develop. MCA is divided into four main portions: proximal M1 occlusion when the entire MCA is infarcted, distal M1 when only the basal ganglia are spared, anterior or posterior trunk or M2 occlusion depending on the damage to frontal or parietal and temporal, respectively, and lenticulostriate when basal ganglia and deep white matter are the only affected parts [2]. Other possible clinical presentations may be IS secondary to venous occlusion; however, this is rare and is associated with other pathologies that will not be discussed in this paper. Due to its low reported incidence, pediatric stroke tends to be frequently undiagnosed or misdiagnosed. In the pediatric population, the average diagnostic time presented goes from 15 hours to three months, which is associated with higher morbidity and mortality rates [4].

Clinical presentation of stroke varies according to the pediatric age group. In groups under one year of age, epileptic seizures and altered mental status may occur more frequently. It is more difficult to detect speech abnormalities and headaches in this group due to minimal expressive speech ability. As age advances, adolescents usually present focal weakness and focal neurological signs such as aphasia, visual alterations, and hemiparesis. Non-specific signs such as fever, nausea/vomiting, and headache usually appear and are not specific to an age group. Due to the multiple etiologies of arterial ischemic stroke, some are more likely to occur in a specific age group and may affect the clinical presentation [5]. Through this case, it was observed that the clinical presentation was compatible with an obstruction at the right MCA, which later was proved in imaging studies. The low prevalence and wide spectrum of etiologies of this pathology can delay diagnosis, therefore arterial IS must be considered in a differential diagnosis for acute-onset neurological deficit. When having a patient with stroke-like symptoms, given the lack of specificity in clinical presentation, neuroimaging is critical, and the preferential imaging modality is MRI. DWI is the most sensitive method. A wide variety of findings can be seen by using DWI but the main characteristic is finding restricted diffusion in an area; this image will appear as an interruption in the motion of water molecules to a specific anatomic location compared to the surrounding areas. Another thing that can be generated from DWI is the ADC map that is used to identify if the ischemic lesion present is acute or subacute (less than seven to ten days). An acute IS is going to present as an area with hyperintensity in DWI with a corresponding hypointensity in ADC. In addition, it has been seen that systemic diseases that cause vasogenic edema, such as preeclampsia, venous thrombosis, and hypertensive encephalopathy, can be differentiated from an IS by using this imaging technique. The best time to perform a DWI on a patient with a suspected ischemic cerebrovascular accident will be between six hours and two days; however, as mentioned earlier, it can also be performed later to differentiate subacute lesions [6]. As seen in this case, hyperacute ischemic vascular disease was identified by using DWI and ADC to establish the temporality of the lesion. Also, the MRI was performed during the first 24 hours from the onset of clinical presentation, which helped to increase the sensibility of the study. It is important to mention that this applies more to adolescents, as, in younger children, different parameters may be used.

Nevertheless, there are pediatric-specific factors that should be considered. Age plays a determinant role when choosing imaging features and imaging protocols, due to the differences in myelination, cerebral blood flow, oxygen metabolism, and neurovascular autoregulation. Another of the main challenges is to achieve high-quality scans in children due to movement artifacts and the future evaluation and availability of sedation/anesthesia [7]. For etiology and to identify vascular abnormalities, vascular imaging of the brain and neck vessels should be considered [6]. As mentioned before, the causes of stroke in the pediatric population are broad and can be divided into multiple categories, most importantly cardiac, extracranial or intracranial arteriopathies, thrombophilia, or systemic causes. In most cases, the cause is multifactorial, which asks for a systematic assessment. Based on clinical suspicion, the examination must be directed but not delayed. Cardiac causes are responsible for almost 30% of pediatric strokes, most of them result from congenital heart disease, commonly PFO. The pathophysiology is mostly thromboembolic. The examination most frequently used is a transthoracic echocardiogram with bubble study and monitorization of the arrhythmia. In this case, the patient underwent a transthoracic echocardiogram, which excluded PFO and showed a normal ejection fraction.

Extracranial arteriopathies can be caused by a craniocervical arterial dissection (CCAD), which is responsible for 7.5% of strokes in pediatric patients. The suggested examinations for detecting a CCAD are magnetic resonance angiography (MRA) and CT angiography unless another entity is found during the initial examination of the causes. Up to 45% of strokes in the pediatric population are caused by intracranial arteriopathies, which has become a standard component when examining childhood strokes. The imaging of the intracranial vessels through the modality of MRA or CTA is also recommended. Other causes of pediatric strokes to take into account when examining are inflammatory and thrombophilic disorders. When no other cause, either structural or cardiac, is found, screening must be considered for both of these and systemic causes with laboratory testing such as C-reactive protein (CRP), antinuclear antibodies (ANA), or erythrocyte sedimentation rate (ESR) [1]. There are no clear specifications for laboratory assessment when examining pediatric stroke causes. Guidelines fail to provide clear indications about the tests that should be requested upon hospital arrival and their relation with the time of onset [8]. Laboratory assessment may include a broad range of nonspecific and more specific tests to search for specific causes, including the aforementioned coagulopathies, systemic causes, or hematological diseases. The laboratory and diagnostic tests that are considered to be useful during the analysis are liver function, ESR, CRP, ANA, lupus anticoagulant, anticardiolipin antibody, beta-2-glycoprotein-1 antibody, activated protein C resistance, factor V Leiden mutation, protein S/C function, antithrombin III, prothrombin gene mutation, homocysteine level, fibrinogen, plasminogen, plasma amino acids, serum lactate/pyruvate, triglycerides, cholesterol, and bacterial/fungal/parasitic/viral/rickettsial tests [4]. This case was approached with a range of nonspecific and specific laboratory tests (Table 1). In this case, the results obtained oriented toward a systemic and autoimmune cause, which was identified after excluding cardiac causes and revealed the formation of a thrombus. Ultimately, the diagnosis of APS was made.

APS, also known as Hughes syndrome, is a systemic autoimmune condition characterized by the occurrence of arterial and/or venous thrombotic events and/or pregnancy morbidity, coupled with the persistent presence of positive antiphospholipid antibodies (aPL). Pediatric APS, specifically diagnosed in individuals under the age of 18 years, typically manifests between nine and 14 years of age [9,10]. Furthermore, it is estimated that up to 24-50% of APS cases in children are classified as primary [11]. As of now, there are no specific criteria established for the diagnosis of APS in children. However, the Sapporo criteria, initially designed for adults, are commonly applied to the pediatric population. These criteria require the presence of at least one clinical event (venous, arterial, small vessel thrombosis, or pregnancy-related morbidity) and the sustained presence for at least 12 weeks of at least one laboratory feature: positive lupus anticoagulant (functional assay screening for aPL), anticardiolipin IgG or IgM in medium or high titer (>40 GPL/MPL or titer > 99th percentile), or anti-beta-2 glycoprotein I (β2GPI) IgG or IgM (titer > 99th percentile) [9]. Common clinical manifestations of APS in children include a range of neurological and hematological symptoms, amongst others. Neurologically, patients may experience headaches, choreiform movements, migraines, pseudotumor cerebri, and conduct disorders. Hematological manifestations can involve thrombocytopenia, which presents with petechiae or ecchymosis and autoimmune hemolytic anemia.

The combination of autoimmune hemolytic anemia with thrombocytopenia is known as “Evans syndrome,” occurring in approximately 10-15% of cases and is associated with high IgM and IgG anticardiolipin antibodies. Venous thrombosis is a prevalent occurrence, affecting approximately 60% of cases. However, arterial thrombosis (most commonly presented as ischemic cardiovascular disease) and small vessel thrombosis may also be present [10,11]. Skin-related symptoms include livedo reticularis, Raynaud's phenomenon, skin ulcers, and pseudo-vasculitic lesions [11,12]. In addition, some cardiac manifestations, such as valvular disease, as well as kidney diseases, including end-stage renal disease and primary adrenocortical insufficiency secondary to adrenal infarction, are noted. Furthermore, the primary symptoms observed in the patient were neurological, including hemiparesis on the left side of the face and body, drooping of the left eye, difficulty in articulation (dysarthria), restricted movement, and a loss of control over urinary sphincter function. When diagnosing pediatric APS, it is recommended to assess for lupus anticoagulant, anticardiolipin IgG and IgM, and anti-β2GPI IgG and IgM. Laboratory findings revealed the absence of hematologic abnormalities, such as hemolytic anemia or thrombocytopenia. Electrolyte levels were within the normal range, and inflammatory markers such as ESR and procalcitonin were also normal. Renal function showed no abnormalities. While lupus anticoagulant tested positive, cardiolipin antibodies IgM and IgG as well as anti-beta-2 glycoprotein IgM and IgG were negative.

Treatment should always be individualized. If venous thrombosis is present and aPL is positive, long-term anticoagulation is recommended, usually starting with low molecular weight heparin or unfractionated heparin. Subsequently, a transition to vitamin K antagonists, such as warfarin, is commonly used for long-term anticoagulation, with a target INR range of 2-3. Moreover, some studies have shown hydroxychloroquine to have an anti-thrombotic effect in APS [13]. For primary and secondary prevention in the presence of aPL, low-dose aspirin is recommended. In the initial presentation of this case, a positive lupus anticoagulant was detected, leading to the decision to initiate treatment with enoxaparin. However, for long-term management, acenocoumarol was ultimately chosen, in addition to aspirin and hydroxychloroquine. Further tests were conducted several weeks after the initiation of treatment, which revealed negative results for all diagnostic indicators for APS. However, some studies have shown that treatment with vitamin K antagonists, such as low molecular weight heparin and warfarin, may lead to false‐negative or false‐positive lupus anticoagulant findings. Therefore, the interdisciplinary team decided to continue treating the patient as APS [14,15].

## Conclusions

Pediatric IS is rare and it is crucial to recognize it early. DWI is preferred for its sensitivity in detecting early changes and distinguishing acute from subacute lesions. A thorough evaluation by rheumatology and hematology teams is essential to rule out systemic causes. Cases like these highlight rare initial signs of complex diseases. Hypercoagulative states, especially APS, should be considered in adolescents with IS. Prompt diagnosis enables early anticoagulation therapy, typically with low molecular weight heparin or unfractionated heparin, transitioning to vitamin K antagonists for long-term management. Interdisciplinary collaboration improves patient outcomes, especially in conditions often associated with high risk. Follow-up at 12 weeks is crucial to confirm APS diagnosis.

## References

[1] Ferriero DM, Fullerton HJ, Bernard TJ (2019). Management of stroke in neonates and children: a scientific statement from the American Heart Association/American Stroke Association. Stroke.

[2] Cárdenas JF, Rho JM, Kirton A (2011). Pediatric stroke. Childs Nerv Syst.

[3] Leal Rato M, Bandeira M, Romão VC, Aguiar de Sousa D (2021). Neurologic manifestations of the antiphospholipid syndrome - an update. Curr Neurol Neurosci Rep.

[4] Tsze DS, Valente JH (2011). Pediatric stroke: a review. Emerg Med Int.

[5] Zimmer JA, Garg BP, Williams LS, Golomb MR (2007). Age-related variation in presenting signs of childhood arterial ischemic stroke. Pediatr Neurol.

[6] Jordan LC, Hillis AE (2011). Challenges in the diagnosis and treatment of pediatric stroke. Nat Rev Neurol.

[7] Sotardi ST, Alves CA, Serai SD, Beslow LA, Schwartz ES, Magee R, Vossough A (2023). Magnetic resonance imaging protocols in pediatric stroke. Pediatr Radiol.

[8] Janes F, Giacomello R, Blarasin F (2022). Contribution and effectiveness of laboratory testing in the diagnostic assessment of juvenile ischemic stroke and transient ischemic attack. Cureus.

[9] Madison JA, Gockman K, Hoy C, Tambralli A, Zuo Y, Knight JS (2022). Pediatric antiphospholipid syndrome: clinical features and therapeutic interventions in a single center retrospective case series. Pediatr Rheumatol Online J.

[10] Rosina S, Chighizola CB, Ravelli A, Cimaz R (2021). Pediatric antiphospholipid syndrome: from pathogenesis to clinical management. Curr Rheumatol Rep.

[11] Torres-Jimenez AR, Ramirez-Nova V, Cespedes-Cruz AI, Sanchez-Jara B, Velazquez-Cruz A, Bekker-Méndez VC, Guerra-Castillo FX (2022). Primary antiphospholipid syndrome in pediatrics: beyond thrombosis. Report of 32 cases and review of the evidence. Pediatr Rheumatol Online J.

[12] Madison JA, Zuo Y, Knight JS (2020). Pediatric antiphospholipid syndrome. Eur J Rheumatol.

[13] Soybilgic A, Avcin T (2020). Pediatric APS: state of the art. Curr Rheumatol Rep.

[14] Favaloro EJ, Pasalic L (2022). Lupus anticoagulant testing during anticoagulation, including direct oral anticoagulants. Res Pract Thromb Haemost.

[15] Tripodi A, Cohen H, Devreese KM (2020). Lupus anticoagulant detection in anticoagulated patients. Guidance from the Scientific and Standardization Committee for lupus anticoagulant/antiphospholipid antibodies of the International Society on Thrombosis and Haemostasis. J Thromb Haemost.

